# Demographic and clinical factors associated with the distance traveled by people with oral cancer: a cross-sectional study, North and Northeast regions of Brazil, 2017-2023

**DOI:** 10.1590/S2237-96222026v35e20250749.en

**Published:** 2026-06-19

**Authors:** Danielle Karla Vieira e Silva, Rênnis Oliveira da Silva, Luciana Leônia Soares Freire, Maria Letícia Barbosa Raymundo, Lilian Nadja Silva Brito, Yuri Wanderley Cavalcanti

**Affiliations:** 1 Universidade Federal da Paraíba Programa de Pós-Graduação em Odontologia João Pessoa PB Brazil Universidade Federal da Paraíba, Programa de Pós-Graduação em Odontologia, João Pessoa, PB, Brazil

**Keywords:** Mouth Neoplasms, Access to Health Services, Health Inequities, Social Conditions, Cross-Sectional Studies, Neoplasias de la Boca, Acceso a los Servicios de Salud, Inequidades en Salud, Condiciones Sociales, Estudios Transversales

## Abstract

**Objective:**

To analyze the demographic and clinical factors associated with the distance traveled by people with oral cancer to get hospital treatment in the North and Northeast regions of Brazil, between 2017 and 2023.

**Methods:**

This was a cross-sectional study conducted with data from the Hospital Cancer Registry. The dependent variable was defined as the distance, in kilometers, between the municipality of residence and the place of hospital admission, measured by OpenStreetMap. The independent variables included the staging of oral cancer (I, II, III or IV), the region (North or Northeast) and the year of treatment. Gamma regression with logarithmic link function was used to estimate ratios of means (RM) and 95% confidence intervals (95%CI).

**Results:**

4,887 records were included. The median distance traveled to treatment was greater in the Northern region (70.9 km). Multivariate analysis revealed significant association between stage IV and greater travel distances (OR 1.05; 95%CI 1.00; 1.11). People from the Northern region (OR 1.38; 95%CI 1.26; 1.51) traveled greater distances to a hospital. A significant increase was observed in 2020 (OR 1.10; 95%CI 1.01; 1.19) and 2022 (OR 1.11; 95%CI 1.02; 1.20).

**Conclusion:**

The greatest distances for hospital treatment of oral cancer occurred among people with advanced stages of the disease residing in the Northern region, highlighting inequalities in access to oncological care.

## Resumen

**Objetivo:**

Analizar los factores demográficos y clínicos asociados a la distancia recorrida por personas con cáncer oral para recibir tratamiento hospitalario en las regiones Norte y Noreste de Brasil, entre 2017 y 2023.

**Métodos:**

Estudio transversal realizado con datos del Registro Hospitalario de Cáncer. La variable dependiente se definió como la distancia, en kilómetros, entre el municipio de residencia y el lugar de ingreso hospitalario, medida mediante OpenStreetMap. Las variables independientes incluyeron el estadio del cáncer oral (I, II, III o IV), la región (Norte o Noreste) y el año de tratamiento. Se utilizó regresión gamma con función de enlace logarítmica para estimar la razón de medias (RM) y el intervalo de confianza del 95% (IC95%).

**Resultados:**

Se incluyeron 4.887 registros. La mediana de la distancia recorrida para recibir tratamiento fue mayor en la región Norte (70,9 km). El análisis multivariante reveló una asociación significativa entre el estadio IV y mayores distancias de viaje (OR 1,05; IC95% 1,00; 1,11). Las personas de la región norte (OR 1,38; IC95%: 1,26; 1,51) recorrieron mayores distancias para llegar al hospital. Se observó un aumento significativo en 2020 (OR 1,10; IC95%: 1,01; 1,19) y en 2022 (OR 1,11; IC95%: 1,02; 1,20).

**Conclusión:**

Las mayores distancias para el tratamiento hospitalario del cáncer oral se observaron entre las personas con estadios avanzados de la enfermedad que residen en la región norte, lo que pone de manifiesto las desigualdades en el acceso a la atención oncológica.

**Ethical aspects**

This research used public domain anonymized databases.

## Introduction

Oral cancer, mostly represented by squamous cell carcinoma, is a considerable global public health problem, related to high morbidity and mortality and significant impairment of quality of life. International estimates indicate approximately 370,000 new cases and 199,000 deaths from lip and oral cavity cancer in more than 200 countries in 2019, and approximately 377,000 cases in 2020, with a higher concentration in Asian countries [1-3].

Incidence and mortality vary significantly according to sex, age group and socioeconomic level, being higher in nations with low and middle human development indices [1,4]. In addition to the clinical burden, cancer imposes substantial financial consequences for people affected by this disease and their families, which exacerbates inequalities and compromises continuous access to treatment [5,6].

International studies have shown that distance to treatment centers is a significant barrier, associated with late diagnoses, poorer clinical outcomes, and higher risk of treatment dropout, as observed in the United States and India in 2024 [7,8]. Among United States citizens with verrucous carcinoma of the oral cavity, greater distances traveled to hospital were related to postoperative radiotherapy being indicated, without a positive impact on survival, suggesting geographical influence on clinical decisions [9].

In Brazil, oral cancer has a high morbidity and mortality burden, with estimates of 15,000 new cases between 2023 and 2025, predominantly among males [10]. Mortality is unevenly distributed across the Brazilian territory, with a higher concentration in socially vulnerable areas [11]. Most cases correspond to squamous cell carcinoma, which mainly affects the lip, tongue and floor of the mouth [12,13].

Investigations conducted in Brazil, including a study carried out in the Rio de Janeiro Metropolitan Region between 2021 and 2022, indicated that social determinants, such as low education levels and unfavorable socioeconomic conditions, contribute to diagnosis at more advanced stages. This reflects persistent inequalities in timely access to cancer diagnosis and treatment [14].

Even with the expansion of the Brazilian Unified Health System (*Sistema Único de Saúde*, SUS), with its primary care and dental specialty center network, barriers to access still persist in areas of greater vulnerability [15-17]. Among these barriers, geographical distance to referral centers stands out as a relevant challenge, especially in the context of territorial inequalities and difficulties faced in rural and remote regions [18-20].

A comparison between the periods 2009-2010 and 2017-2018 found that 49% and 61% of SUS service users, respectively, underwent cancer treatment outside their municipality of residence, with the greatest distances to treatment recorded in the North and Midwest regions of the country, ranging from 296 km to 870 km [19]. These findings emphasized the need for regional organization of the oncology network, with a view to reducing geographical barriers and promoting territorial equity [19].

The influence of distance on access to cancer treatment is a factor worthy of attention: between 2019 and 2020, most women with breast cancer started therapy more than 60 days after diagnosis, with even longer intervals among those who traveled outside their municipality, covering average distances close to 300 km [21]. These results highlighted that both the distance and location of health services are significant obstacles to timely treatment. This converges with findings resulting from the analysis of geographical accessibility focusing on cancer service users as a whole in Brazil [19].

The epidemiological profile and survival of people with head and neck tumors have already been described in Brazil [22]. With regard to oral cancer, however, few studies have investigated territorial and socioeconomic factors associated with access to treatment [19,21]. Measuring the distance traveled by people with oral cancer helps to identify regional inequalities in access to treatment on the SUS.

This study aimed to analyze the demographic and clinical factors associated with the distance traveled by SUS service users with oral cancer to hospital treatment in the North and Northeast regions of Brazil, between 2017 and 2023.

## Methods

### Design 

This is an observational and cross-sectional study. 

### Setting

This study used records of patients with oral cancer residing in the North and Northeast regions of Brazil, treated in SUS hospital units, between 2017 and 2023. The analysis of the distance between the municipality of residence and the place of treatment aimed to describe travel patterns and compare regional variations in the distribution of oncology services, in order to support the planning of the healthcare network.

### Participants 

The study population consisted of records of individuals with confirmed diagnosis of oral cancer, corresponding to topographic sites C00 to C06, as per the 10th revision of the International Classification of Diseases (ICD-10).

Initially, 6,816 records related to oral neoplasms were identified in the two regions during the period analyzed. No duplicate records were identified. 1,929 records were excluded due to essential information being missing, such as municipality of residence, treatment hospital unit, or clinical staging. Once these had been excluded, 4,887 records remained eligible and comprised the final database used in the statistical analyses.

### Variables

The dependent variable was the distance traveled (in kilometers) between the service user’s municipality of residence and the municipality in which the hospital unit where the treatment took place was located. This variable was expressed as a continuous value, and, for cases where people resided in the same municipality as the hospital where they were treated, a value of 1 km was assigned, in accordance with the statistical model prerequisite.

The independent variables were: geographic region (North and Northeast), clinical staging (I, II, III and IV) and year of care (2017 to 2023).

### Data source 

The data were obtained from the Hospital Cancer Registry, provided by the National Cancer Institute and accessed on September 10, 2024. We used information regarding municipality of residence, hospital in which treatment was provided and clinical staging.

Distance traveled was estimated between the geographic centers (centroids) of the municipalities of residence and treatment. The origin and destination coordinates were used in an Open Source Routing Machine service, accessed through the OpenStreetMap interface, with a car travel profile, estimating the shortest route (in kilometers) along the available road network. Therefore, the estimated distance does not correspond to the Euclidean distance, but rather to the route calculated on the mapped transport network. In regions with less road connectivity, such as areas in the Northern region, the estimated distance reflected the transport infrastructure available in the cartographic database.

### Bias

As the data was derived from the Hospital Cancer Registry, there is a possibility of incomplete coverage due to some hospitals not being included in the Registry, as well as the possibility of exclusion of records with missing information. In order to mitigate these biases, rigorous inclusion criteria were applied, and double-checking of data extraction and consistency was performed. Furthermore, geographic distance was estimated from the geographic centers (centroids) of the municipalities, which may not accurately reflect the actual distance traveled by users, especially in areas with less transport connectivity. In cases where the municipality of residence coincided with the municipality of treatment, a minimum value of 1 km was assigned, a procedure adopted exclusively to enable statistical modeling.

### Sample size 

All eligible records of individuals with confirmed diagnosis of oral cancer held on in the Hospital Cancer Registry for the period analyzed were included.

### Statistical methods

The data were organized on Microsoft Excel 2019 spreadsheets and analyzed using Jamovi 2.6 software. Descriptive analysis was used to characterize the study population, presenting absolute and relative frequencies for categorical variables and measures of central tendency and dispersion for the continuous variable (distance traveled in kilometers). In the inferential analysis, the gamma regression model with a logarithmic link function was applied, appropriate for continuous and positive variables, such as distance. This approach is recommended for asymmetrical data because it generates consistent estimates [23].

We estimated ratios of means (RM) and their respective 95% confidence intervals (95%CI), taking a 5% significance level. As a prerequisite for the model, the distance traveled by people residing in the same municipality as the hospital unit was adjusted to a value of 1 km.

## Results

A total of 4,887 records of people with oral cancer in the North and Northeast regions of Brazil between 2017 and 2023 were analyzed (Table 1). Characterization of the sample revealed a predominance of people from the Northeast (n=4,456; 91.2%) and people living in interior region municipalities (n=3,320; 68.0%). The majority of service users were diagnosed at advanced stages (IV: 57.0%), while 43.0% were in the initial stages (I-III). Annual distribution showed a higher concentration of records in 2017 (23.7%) and 2018 (22.0%) (Table 1).

**Table 1:** Descriptive analysis of the distance traveled in kilometers (km) by people with oral cancer, according to clinical staging, region, type of municipality and year of treatment. North and Northeast regions of Brazil, 2017-2023 (n=4,887)

		Distance (km)		
Variables	n (%)	Median	Q1^a^	Q3^b^
Region				
Northeast	4,456 (91.2)	61.0	1	241
North	431 (9.0)	70.9	1	228
**Cancer staging **(**4 versus 1**-3)				
I-III	2,102 (43.0)	58.0	1	232
IV	2,785 (57.0)	63.3	1	244
**Type of municipality**				
State capital	1,567 (32.0)	1.0	1	1
Interior region	3,320 (68.0)	158.0	58	329
Year				
2017	1,160 (23.7)	55.5	1	222
2018	1,053 (22.0)	62.3	1	242
2019	823 (16.9)	64.3	1	240
2020	703 (14.4)	72.1	1	247
2021	203 (4.2)	55.3	3	180
2022	843 (17.6)	52.6	1	254
2023	102 (2.1)	78.5	1	251

^a^Q1: first quartile; ^b^Q3: third quartile.

The median distance traveled to treatment varied according to clinical and geographic characteristics: 58.0 km in stages I-III and 63.3 km in stage IV; 70.9 km among those living in the Northern region, this being higher than the 61.0 km observed in the Northeast. People living in interior region municipalities traveled a median of 158.0 km, while those living in state capitals traveled 1.0 km (minimum value adopted to enable the gamma regression model) (Table 1).

The gamma regression results, using a logarithmic link function, showed that advanced clinical staging (IV) of service users was associated with a 5% increase in average distance traveled, when compared to stages I-III (RM 1.05; 95%CI 1.00; 1.11; p-value 0.047). Northern region residents needed to travel 38.0% greater distances than Northeast region residents (RM 1.38; 95%CI 1.26; 1.51; p-value<0.001). Among the years analyzed, significant increases were observed in 2020 and 2022.

The variation in average distance (in kilometers) traveled by people in the North and Northeast regions, stratified by clinical staging, between 2017 and 2023 is presented in Figure 1. In the Northeast, the values ​​ranged from 12.0 km to 16.5 km, while in the North they were between 17.5 km and 22.5 km.

**Figure 1:**
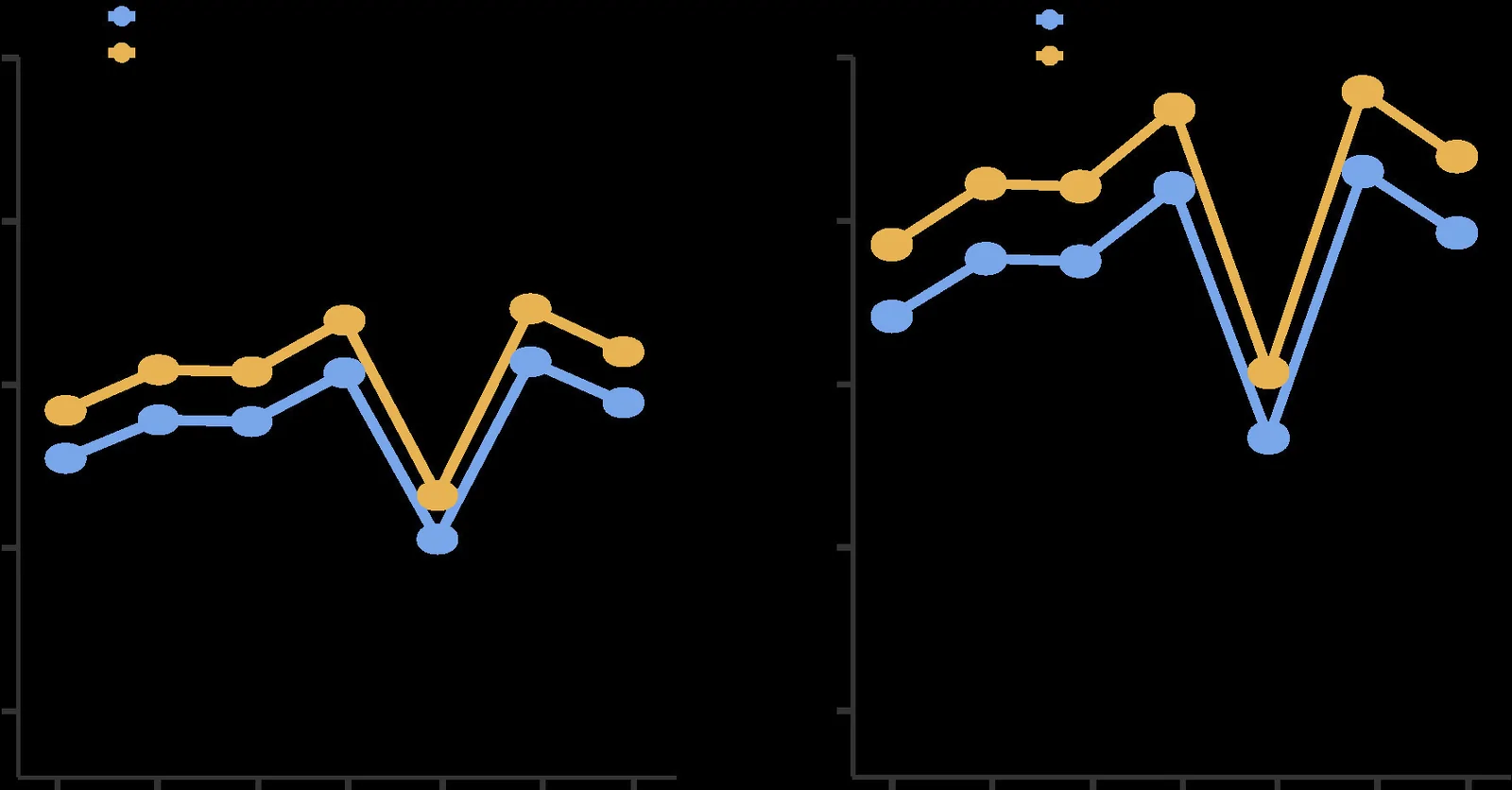
Distance traveled (km) between residence and place of treatment, by stage (I-III and IV) and year: (A) Northeast; (B) North. Northeast and Northern Regions of Brazil, 2017-2023 (n=4,887)

## Discussion

This study demonstrated that factors such as advanced clinical staging and being located in Northern Brazil were associated with greater distances traveled to referral hospital units. These findings indicated that territorial and socioeconomic inequalities still restrict timely access to cancer care, despite structural progress achieved by the SUS. Concentration of specialized services in larger urban centers imposes longer traveling distances for people who live in rural and inner state areas, which can lead to delays in diagnosis and treatment only being started when clinical conditions are already more severe.

Standing out among the limitations of this study is the incomplete coverage of the Hospital Cancer Registry, since not all hospitals are included on it. Moreover, records with missing information were excluded. There was also the possibility of measurement bias, as the distance was calculated between city centers, which did not represent the actual route taken by service users. Furthermore, in order to enable gamma regression, a value of 1 km was assigned to people who lived in the same municipality as the hospital where they were treated, which may not accurately reflect these cases.

Another possible bias was case severity, since service users at clinical stage IV tend to be referred to more complex centers, generally further away. Finally, the absence of socioeconomic variables, such as income, limited the possibility of fully adjusting for confounders. Despite these points, the geographic scope and sample size provided the results with consistency and relevance.

The prevalence of service users in the Northeast can be explained by this region’s population size and high proportional oral cancer burden [19,13]. In the North, distances were longer due to the continental dimensions of this region, predominance of river transport and scarcity of hospital units in the interior, factors that create gaps in care and require longer journeys [19,24]. Centralization of services in state capitals exacerbates this situation, since maintaining oncology centers and establishing multidisciplinary teams in remote areas is costly and difficult to implement [19].

The fluctuations observed over the years in distances traveled, especially the increases in 2020 and 2022 identified in the regression analysis, suggest structural impacts on the supply and demand for cancer care. These patterns are consistent with the literature describing temporary disruptions in the network during the COVID-19 pandemic and the subsequent resumption of care, often with people in more advanced stages, which may require referral to more distant centers [25]. The greater distance traveled by stage IV service users and those living in the North further emphasizes that clinical and territorial factors interact in determining inequalities in access, converging with previous findings in countries with large regional disparities [7,18].

This study highlighted that geographical distance is a significant barrier to oral cancer treatment in the North and Northeast regions of Brazil, which demonstrates territorial inequalities that require regional planning and strengthening of the oncology network.

## Data Availability

The data used in this study are publicly available and were obtained from the Hospital Cancer Registry, made available by the José Alencar Gomes da Silva National Cancer Institute, through the RHCNet platform (https://irhc.inca.gov.br/RHCNet/).
