# Supplementary material for: Demographic and clinical factors associated with the distance traveled by people with oral cancer: a cross-sectional study, North and Northeast regions of Brazil, 2017-2023
Source: Epidemiol Serv Saude. 2026 Jun 19;35:e20250749. doi: 10.1590/S2237-96222026v35e20250749.en (PMC13686296; doi:10.1590/S2237-96222026v35e20250749.en)
Supplement: Translation

# Translation: Fatores demográficos e clínicos associados à distância percorrida por pessoas com câncer de boca: estudo transversal, regiões Norte e Nordeste, 2017-2023

**DOI:** 10.1590/S2237-96222026v35e20250749.pt

**Authors:** Danielle Karla Vieira e Silva, Rênnis Oliveira da Silva, Luciana Leônia Soares Freire, Maria Letícia Barbosa Raymundo, Lilian Nadja Silva Brito, Yuri Wanderley Cavalcanti

**Affiliations:** 1 Universidade Federal da Paraíba Programa de Pós-Graduação em Odontologia João Pessoa PB Brasil Universidade Federal da Paraíba, Programa de Pós-Graduação em Odontologia, João Pessoa, PB, Brasil

**Keywords:** Neoplasias Bucais, Acesso aos Serviços de Saúde, Disparidades em Saúde, Condições Sociais, Estudos Transversais

## Abstract

**Objetivo:**

Analisar os fatores demográficos e clínicos associados à distância percorrida por pessoas com câncer de boca até o tratamento hospitalar, nas regiões Norte e Nordeste do Brasil, entre 2017 e 2023.

**Métodos:**

Estudo transversal realizado com dados do Registro Hospitalar de Câncer. Definiu-se como variável dependente a distância, em quilômetros, entre o município de residência e o local de internação hospitalar, mensurada pelo OpenStreetMap. As variáveis independentes incluíram o estadiamento do câncer de boca (I, II, III ou IV), a região (Norte ou Nordeste) e o ano de atendimento. A regressão gama com função de ligação logarítmica foi empregada para estimar a razão de média (RM) e o intervalo de confiança de 95% (IC95%).

**Resultados:**

Foram incluídos 4.887 registros. A mediana da distância percorrida até o tratamento foi maior na região Norte (70,9 km). A análise multivariada revelou associação significativa entre o estadiamento IV e deslocamentos maiores (RM 1,05; IC95% 1,00; 1,11). Pessoas da região Norte (RM 1,38; IC95% 1,26; 1,51) percorreram maiores distâncias até o hospital. Observou-se aumento significativo em 2020 (RM 1,10; IC95% 1,01; 1,19) e 2022 (RM 1,11; IC95% 1,02; 1,20).

**Conclusão:**

As maiores distâncias para o tratamento hospitalar do câncer de boca ocorreram entre pessoas em estágio avançado da doença, residentes da região Norte, o que evidenciou desigualdades no acesso ao cuidado oncológico.

**Aspectos éticos**

Esta pesquisa utilizou bancos de dados de domínio público e anonimizados.

## Introdução

O câncer de boca, em sua maioria representado pelo carcinoma espinocelular, configura expressivo problema de saúde pública mundial, relacionado a alta morbimortalidade e prejuízos significativos à qualidade de vida. Estimativas internacionais apontam cerca de 370 mil novos casos e 199 mil óbitos por câncer de lábio e cavidade oral em mais de 200 países em 2019, e aproximadamente 377 mil casos em 2020, com maior concentração em países asiáticos [1-3]. 

A incidência e a mortalidade variam de forma expressiva segundo sexo, faixa etária e nível socioeconômico, sendo mais elevadas em nações de baixo e médio índice de desenvolvimento humano [1,4]. Além do ônus clínico, o câncer impõe consequências financeiras substanciais para as pessoas acometidas por essa enfermidade e para as suas famílias, o que agrava desigualdades e compromete o acesso contínuo ao tratamento [5,6].

Estudos internacionais demonstraram que a distância até os centros de tratamento constitui uma barreira relevante, associada a diagnósticos tardios, piores desfechos clínicos e maior risco de abandono terapêutico, conforme observado nos Estados Unidos e na Índia, em 2024 [7,8]. Em norte-americanos com carcinoma verrucoso de cavidade oral, distâncias maiores percorridas até o hospital estiveram relacionadas à indicação de radioterapia pós-operatória, sem impacto positivo na sobrevida, sugerindo influência geográfica nas decisões clínicas [9].

No Brasil, o câncer de boca apresenta elevada carga de morbimortalidade, com estimativas de 15 mil novos casos no triênio 2023-2025, predominando entre os homens [10]. A mortalidade é desigualmente distribuída no território brasileiro, com maior concentração em áreas socialmente vulneráveis [11]. A maioria dos casos corresponde ao carcinoma espinocelular, que acomete principalmente lábio, língua e assoalho bucal [12,13]. 

Investigações conduzidas no país, incluindo estudo desenvolvido na Região Metropolitana do Rio de Janeiro entre 2021 e 2022, indicaram que determinantes sociais, como baixa escolaridade e condições socioeconômicas desfavoráveis, contribuem para o diagnóstico em estágios mais avançados. Isso reflete desigualdades persistentes no acesso oportuno ao diagnóstico e ao tratamento oncológico [14].

Mesmo com a ampliação do Sistema Único de Saúde (SUS), com a atenção primária e a rede de centros de especialidades odontológicos, ainda persistem barreiras de acesso em áreas de maior vulnerabilidade [15-17]. Entre essas barreiras, a distância geográfica até os centros de referência se destaca como desafio relevante, especialmente no contexto das desigualdades territoriais e das dificuldades enfrentadas em regiões rurais e remotas [18-20].

Na comparação entre os períodos 2009-2010 e 2017-2018, foi demonstrado que 49% e 61% dos usuários do SUS, respectivamente, realizaram o tratamento de câncer fora de seu município de residência, com maiores distâncias registradas nas regiões Norte e Centro-Oeste, variando entre 296 km e 870 km [19]. Esses achados reforçaram a necessidade de organização regional da rede oncológica, com vistas à redução das barreiras geográficas e à promoção da equidade territorial [19].

A influência da distância no acesso ao tratamento oncológico é fator a ser destacado: entre 2019 e 2020, a maioria das mulheres acometidas por câncer de mama iniciou a terapia após mais de 60 dias do diagnóstico, com intervalos ainda maiores entre aquelas que se deslocaram para fora do município, percorrendo médias próximas de 300 km [21]. Tais resultados reforçaram que tanto a distância quanto a localização dos serviços de saúde configuram obstáculos relevantes à oportunidade terapêutica. Isso converge com achados da análise de acessibilidade geográfica voltada ao conjunto de usuários oncológicos no país [19].

O perfil epidemiológico e a sobrevida de pessoas com tumores de cabeça e pescoço já foram descritos no Brasil [22]. No câncer de boca, entretanto, poucos estudos têm investigado os fatores territoriais e socioeconômicos associados ao acesso ao tratamento [19,21]. A mensuração da distância percorrida por pessoas com câncer de boca contribui para identificar desigualdades regionais de acesso ao tratamento no SUS.

Este estudo teve como objetivo analisar os fatores demográficos e clínicos associados à distância percorrida por usuários com câncer de boca até o tratamento hospitalar, nas regiões Norte e Nordeste do Brasil, entre 2017 e 2023.

## Métodos

### Delineamento do estudo 

Trata-se de estudo observacional e transversal.

### Contexto

O estudo utilizou registros de usuários com câncer de boca residentes nas regiões Norte e Nordeste do Brasil, atendidos em unidades hospitalares vinculadas ao SUS, no período 2017-2023. A análise da distância entre o município de residência e o local de tratamento teve como objetivo descrever padrões de deslocamento e comparar variações regionais na distribuição dos serviços oncológicos, com vistas a subsidiar o planejamento da rede assistencial.

### Participantes 

A população do estudo foi composta por registros de pessoas com diagnóstico confirmado de câncer de boca, correspondentes às localizações topográficas C00 a C06, segundo a 10ª revisão da Classificação Internacional de Doenças (CID-10).

Inicialmente, foram identificados 6.816 registros referentes a neoplasias de boca nas duas regiões no período analisado. Não foram identificados registros duplicados. Foram excluídos 1.929 registros devido à ausência de informações essenciais, como município de residência, unidade hospitalar de tratamento ou estadiamento clínico. Após as exclusões, 4.887 registros permaneceram elegíveis e compuseram a base final utilizada nas análises estatísticas.

### Variáveis

A variável dependente foi a distância percorrida (em quilômetros) entre o município de residência do usuário e o município da unidade hospitalar onde ocorreu o tratamento. Essa variável foi expressa como valor contínuo, e, para os casos em que as pessoas residiam no mesmo município do hospital, foi atribuído o valor de 1 km, conforme o pré-requisito do modelo estatístico. 

As variáveis independentes foram: região geográfica (Norte e Nordeste), estadiamento clínico (I, II, III e IV) e ano do atendimento (2017 a 2023).

### Fonte de dados

Os dados foram obtidos do Registro Hospitalar de Câncer, disponibilizado pelo Instituto Nacional de Câncer e acessado em 10 de setembro de 2024. Foram utilizadas as informações referentes ao município de residência, à unidade hospitalar de tratamento e ao estadiamento clínico. 

A distância percorrida foi estimada entre os centros geográficos (centroides) dos municípios de residência e de tratamento. As coordenadas de origem e destino foram utilizadas em um serviço de roteamento viário (Open Source Routing Machine), acessado por meio da interface do OpenStreetMap, com perfil de deslocamento por automóvel, estimando-se a rota de menor distância (em quilômetros) ao longo da rede viária disponível. Dessa forma, a distância estimada não corresponde à distância euclidiana, mas ao percurso calculado na malha de transportes mapeada. Em regiões com menor conectividade rodoviária, como áreas da região Norte, a distância estimada refletiu a infraestrutura de transporte disponível na base cartográfica.

### Viés

Por se tratar de dados provenientes dos Registros Hospitalares de Câncer, há possibilidade de cobertura incompleta, devido à não participação de alguns hospitais, e de exclusão de registros com informações faltantes. Para mitigar esses vieses, aplicaram-se critérios rigorosos de inclusão e procedeu-se à dupla checagem da extração e consistência dos dados. Além disso, a distância geográfica foi estimada a partir dos centros geográficos (centroides) dos municípios, o que pode não refletir com precisão o trajeto efetivamente percorrido pelos usuários, especialmente em áreas com menor conectividade de transporte. Nos casos em que o município de residência coincidiu com o do tratamento, atribuiu-se o valor mínimo de 1 km, procedimento adotado exclusivamente para viabilizar a modelagem estatística.

### Tamanho da amostra

Foram incluídos todos os registros elegíveis de pessoas com diagnóstico confirmado de câncer de boca disponíveis no Registro Hospitalar de Câncer para o período analisado.

### Métodos estatísticos

Os dados foram organizados em planilhas do Microsoft Excel 2019 e analisados no software Jamovi 2.6. A análise descritiva foi utilizada para caracterizar a população do estudo, com apresentação de frequências absolutas e relativas para variáveis categóricas e medidas de tendência central e dispersão para a variável contínua (distância percorrida em quilômetros). Na análise inferencial, aplicou-se o modelo de regressão gama com função de ligação logarítmica, apropriado para variáveis contínuas e positivas, como distância. Essa abordagem é recomendada para dados assimétricos por gerar estimativas consistentes [23].

Foram estimadas razões de médias (RM) e seus respectivos intervalos de confiança de 95% (IC95%), adotando-se nível de significância de 5%. Como pré-requisito do modelo, a distância percorrida por pessoas residentes no mesmo município da unidade hospitalar foi ajustada para o valor de 1 km.

## Resultados

Foram analisados 4.887 registros de pessoas com câncer de boca nas regiões Norte e Nordeste do Brasil, entre 2017 e 2023 (Tabela 1). A caracterização da amostra revelou predominância de pessoas oriundas do Nordeste (n=4.456; 91,2%) e residentes em municípios do interior (n=3.320; 68,0%). A maioria dos usuários foi diagnosticada em estágios avançados (IV: 57,0%), enquanto 43,0% encontravam-se nos estágios iniciais (I-III). A distribuição anual mostrou maior concentração de registros em 2017 (23,7%) e 2018 (22,0%) (Tabela 1).

**Tabela 1:** Análise descritiva da distância percorrida em quilômetros (km) por pessoas com câncer de boca, segundo estadiamento clínico, região, tipo de município e ano de atendimento. Regiões Norte e Nordeste, 2017-2023 (n=4.887)

		Distância (km)		
Variáveis	n (%)	Mediana	Q1^a^	Q3^b^
Região				
Nordeste	4.456 (91,2)	61,0	1	241
Norte	431 (9,0)	70,9	1	228
**Estadiamento do câncer **(**4 versus demais**)				
I a III	2.102 (43,0)	58,0	1	232
IV	2.785 (57,0)	63,3	1	244
**Tipo de cidade**				
Capital	1.567 (32,0)	1,0	1	1
Interior	3.320 (68,0)	158,0	58	329
Ano				
2017	1.160 (23,7)	55,5	1	222
2018	1.053 (22,0)	62,3	1	242
2019	823 (16,9)	64,3	1	240
2020	703 (14,4)	72,1	1	247
2021	203 (4,2)	55,3	3	180
2022	843 (17,6)	52,6	1	254
2023	102 (2,1)	78,5	1	251

^a^Q1: primeiro quartil; ^b^Q3: terceiro quartil.

A mediana da distância percorrida até o tratamento variou conforme as características clínicas e geográficas: 58,0 km nos estágios I-III e 63,3 km no estágio IV; 70,9 km entre residentes do Norte, superior aos 61,0 km observados no Nordeste. Moradores do interior percorreram mediana de 158,0 km, enquanto os das capitais deslocaram-se 1,0 km (valor mínimo adotado para viabilizar o modelo de regressão gama) (Tabela 2).

**Tabela 2:** Razões de médias (RM) e intervalos de confiança de 95% (IC95%) da regressão gama da distância percorrida (km) por pessoas com câncer de boca (C00-C06), segundo fatores clínicos, geográficos e temporais. Regiões Norte e Nordeste, 2017-2023 (n=4.887)

Variável	RM (IC95%)	p-valor
**Estadiamento do câncer**		
Estadiamento IV-Estadiamento I-III	1,05 (1,00; 1,11)	0,047
Região		
Norte-Nordeste	1,38 (1,26; 1,51)	<0,001
Ano		
2018-2017	1,04 (0,97; 1,13)	0,274
2019-2017	1,04 (0,96; 1,13)	0,330
2020-2017	1,10 (1,01; 1,19)	0,035
2021-2017	0,91 (0,80; 1,04)	0,169
2022-2017	1,11 (1,02; 1,20)	0,012
2023-2017	1,06 (0,89; 1,28)	0,519

Nos resultados da regressão gama, com função de ligação logarítmica, o estadiamento clínico avançado (IV) dos usuários foi associado ao aumento de 5% na distância média percorrida, quando comparado aos estadiamentos I-III (RM 1,05; IC95% 1,00; 1,11; p-valor 0,047). Residentes do Norte precisaram percorrer distâncias 38,0% maiores que residentes do Nordeste (RM 1,38; IC95% 1,26; 1,51; p-valor<0,001). Entre os anos analisados, observaram-se aumentos significativos em 2020 e 2022. 

Foi apresentada a variação da distância média (em quilômetros) percorrida por pessoas nas regiões Norte e Nordeste, estratificada por estadiamento clínico, entre 2017 e 2023 (Figura 1). No Nordeste, os valores variaram entre 12,0 km e 16,5 km, enquanto no Norte permaneceram entre 17,5 km e 22,5 km. 

**Figura 1:** Distância percorrida (km) entre residência e tratamento, por estadiamento (I-III e IV) e ano: (A) Nordeste; (B) Norte. Regiões Nordeste e Norte, 2017-2023 (n=4.887)

## Discussão

O estudo demonstrou que fatores como o estadiamento clínico avançado e a localização no Norte do Brasil se associaram a maiores distâncias percorridas até unidades hospitalares de referência. Esses achados indicaram que desigualdades territoriais e socioeconômicas ainda restringem o acesso oportuno ao cuidado oncológico, mesmo após avanços estruturais do SUS. A concentração de serviços especializados em centros urbanos de maior porte impõe deslocamentos mais longos para pessoas de áreas rurais e interioranas, o que pode repercutir em atraso diagnóstico e início do tratamento em condições clínicas mais graves. 

Entre as limitações do estudo, destacaram-se a cobertura incompleta do Registro Hospitalar de Câncer, uma vez que nem todos os hospitais participam, e a exclusão de registros com informações faltantes. Houve também a possibilidade de viés de aferição, pois a distância foi calculada entre as sedes dos municípios, o que não representou o trajeto real dos usuários. Além disso, para viabilizar a regressão gama, atribuiu-se o valor de 1 km aos residentes no mesmo município do hospital, o que pode não refletir fielmente esses casos. 

Outro possível viés foi o de severidade, já que usuários em estágio clínico IV tendem a ser encaminhados para centros de maior complexidade, geralmente mais distantes. Por fim, a ausência de variáveis socioeconômicas, como renda, limitou a possibilidade de ajustar completamente os confundidores. Apesar desses pontos, a abrangência geográfica e o tamanho da amostra conferiram consistência e relevância aos resultados.

A prevalência de usuários do Nordeste pode ser explicada pelo tamanho populacional da região e pela elevada carga proporcional de câncer de boca [19,13]. No Norte, as distâncias foram mais longas devido às dimensões continentais, à predominância do transporte fluvial e à escassez de unidades no interior, fatores que criam vazios assistenciais e obrigam deslocamentos maiores [19,24]. A centralização dos serviços nas capitais agrava esse quadro, já que a manutenção de centros oncológicos e a fixação de equipes multiprofissionais em áreas remotas são de alto custo e difícil execução [19].

As oscilações observadas nas distâncias percorridas ao longo dos anos, especialmente os aumentos em 2020 e 2022 identificados na análise de regressão, sugerem impactos estruturais na oferta e na busca por cuidados oncológicos. Esses padrões são compatíveis com a literatura que descreve desorganizações temporárias da rede durante a pandemia de covid-19 e a posterior retomada de atendimentos, frequentemente com pessoas em estágios mais avançados, o que pode demandar encaminhamento para centros mais distantes [25]. A maior distância percorrida por usuários em estadiamento IV e residentes do Norte reforça, ainda, que fatores clínicos e territoriais interagem na determinação das desigualdades de acesso, convergindo com achados prévios em países com grandes disparidades regionais [7,18].

Este estudo destacou que a distância geográfica constitui barreira significativa ao tratamento do câncer de boca nas regiões Norte e Nordeste do Brasil, o que evidencia desigualdades territoriais que exigem planejamento regional e fortalecimento da rede oncológica.

## Data Availability

Os dados utilizados neste estudo são de acesso público e foram obtidos do Registro Hospitalar de Câncer, disponibilizado pelo Instituto Nacional de Câncer José Alencar Gomes da Silva, por meio da plataforma RHCNet (https://irhc.inca.gov.br/RHCNet/).
